# Quantitative ColourDopplerSonography Evaluation of Cerebral Venous Outflow: A Comparative Study between Patients with Multiple Sclerosis and Controls

**DOI:** 10.1371/journal.pone.0025012

**Published:** 2011-09-22

**Authors:** Lucia Monti, Elisabetta Menci, Monica Ulivelli, Alfonso Cerase, Sabina Bartalini, Pietro Piu, Nicola Marotti, Sara Leonini, Paolo Galluzzi, Daniele G. Romano, Alfredo E. Casasco, Carlo Venturi

**Affiliations:** 1 Unit of Neuroimaging and Neurointervention, Department of Neurological and Sensorial Sciences, Azienda Ospedaliera Universitaria Senese, Santa Maria alle Scotte General Hospital, Siena, Italy; 2 Department of Neurosciences, University of Siena, Siena, Italy; 3 Unit of Endovascular and Percutaneous Therapy, Clinica Nuestra Señora del Rosario, Madrid, Spain; Institute Biomedical Research August Pi Sunyer (IDIBAPS) - Hospital Clinic of Barcelona, Spain

## Abstract

**Background:**

Internal Jugular Veins (IJVs) are the principle outflow pathway for intracranial blood in clinostatism condition. In the seated position, IJVs collapse, while Vertebral Veins (VVs) increase the venous outflow and partially compensate the venous drainage. Spinal Epidural Veins are an additional drainage pathway in the seated position. Colour- Doppler-Sonography (CDS) examination is able to demonstrate IJVs and VVs outflow in different postural and respiratory conditions. The purpose of this study was to evaluate CDS quantification of the cerebral venous outflow (CVF) in healthy subjects and patients with multiple sclerosis (MS).

**Methodology/Principal Findings:**

In a group of 27 healthy adults (13 females and 14 males; mean age 37.8±11.2 years), and 52 patients with MS (32 females and 20 males; mean age 42.6±12.1 years), CVF has been measured in clinostatism and in the seated position as the sum of the flow in IJVs and VVs. The difference between CVF in clinostatism and CVF in the seated position (ΔCVF) has been correlated with patients' status (healthy or MS), and a number of clinical variables in MS patients. Statistical analysis was performed by Fisher's exact test, non-parametric Mann-Whitney U test, ANOVA Kruskal-Wallis test, and correntropy coefficient.

The value of ΔCVF was negative in 59.6% of patients with MS and positive in 96.3% of healthy subjects. Negative ΔCVF values were significantly associated with MS (p**<**0.0001). There was no significant correlation with clinical variables.

**Conclusions/Significance:**

Negative ΔCVF has a hemodynamic significance, since it reflects an increased venous return in the seated position. This seems to be a pathologic condition. In MS patients, a vascular dysregulation resulting from involvement of the autonomous nervous system may be supposed. ΔCVF value should be included in the quantitative CDS evaluation of the cerebral venous drainage, in order to identify cerebral venous return abnormalities.

## Introduction

Total arterial cerebral blood flow (CBF) estimation by means of quantitative Doppler flow volume measurement of the extracranial internal carotid and vertebral arteries has been proposed, and is currently performed.[1]–[3] This methodological approach can be also applied to venous extracranial system. High resolution ultrasound can also demonstrate morphologic aspects of Internal Jugular Veins (IJVs), and Vertebral Veins (VVs) such as presence, absence or incompetence of venous valves [4]–[6] and narrowing or stenosis. IJVs and VVs drain the superficial and the deep cerebral venous system. Postural dependency of the cerebral venous outflow (CVF) has been demonstrated in healthy subjects by Colour-Doppler Sonography (CDS).[7]–[11] Anatomical findings support that IJVs are the principle outflow pathway for intracranial blood in clinostatism condition (0°). IJVs collapse in the seated position, while VVs increase the venous outflow and partially compensate the venous drainage. [7]–[8], [12] In the seated position (90°) Spinal Epidural Veins (SEVs) are probably an additional drainage pathway, [13]–[15] since the rigid bony cage around the epidural space may prevent the veins to collapse. IJVs and VVs outflow can be demonstrated by CDS examination in different postural and respiratory conditions. [10], [13], [16] SEVs can only be shown morphologically by other techniques such as magnetic resonance (MR) imaging, MR Angiography, [17] and digital subtraction angiography and/or flebography. [18] Thus, the cerebral venous drainage may be assumed as a liquid deposit with two main outflow pathways, i.e. IJVs and VVs. Calculating the flow of the distal tract of each of these veins it is possible to obtain the majority of CVF in clinostatism and seated position (0°and 90°). Recently, the literature has identified a new nosologic vascular pattern named as chronic cerebrospinal venous insufficiency (CCSVI) in patients with multiple sclerosis (MS). [18]–[23] This physiopathological assessment is diagnosed by intra- and extracranial venous CDS examinations on the basis of five morphologic and dynamic parameters. [15], [19] This theory suggests a role for venous congestion in the pathogenesis of MS.

MS is an inflammatory/degenerative disease of central nervous system with focal demyelination around cerebral veins. This topographic pattern might be correlated to venous congestion, [21], [24] although different findings have been demonstrated by other authors.[25]–[28] Notably, Baracchini et al. [25] do not support a cause–effect relationship between CCSVI and MS, and Doepp et al. [26] do not confirm the ECD data reported by Zamboni et al. [19]

Our work does not analyse the ultrasonographic parameters reported by Zamboni and does not consider CCSVI as the first step. The aim was to evaluate whether there is a statistically significant difference of CVF in clinostatism and in the seated position between healthy subjects and patients with MS.

## Materials and Methods

This study was approved by the Institutional Review Board of the Ethics Committee of the University of Siena, and a written consent was obtained by all the subjects. 52 patients with MS (32 females and 20 males; mean age 42.6±12.1 years) and 27 age-matched healthy volunteers (13 females and 14 males; mean age 37.8±11.2 years) were enrolled. Patients and healthy subjects underwent neurological assessment and CDS examination.

The patients were divided into the four different subgroups of MS, and the fifth reference group of controls. Group 1 included clinically isolated syndrome (CIS) (n:2), Group 2 included relapsing remitting MS (RRMS) (n: 31), Group 3 included secondary progressive MS (SPMS) (n:17), Group 4 included primary progressive MS (PPMS) (n:2), and Group 5 included controls (n:27). The degree of patients' disability was assessed using the Expanded Disability Status Scale (EDSS), [29] the arm/hand dexterity was tested by nine hole peg test (NHPT) and leg function by timed 8meter walk test (T8) [30] prior to the CDS studies.

CDS was performed by two skilled neuroradiologists (LM, and EM) with experience in ultrasound field by using a colour-coded ultrasound system (SEQUOIA, Siemens, Erlangen, Deutschland) and a 7 to 9 MHz linear probe.

The first consecutive 30 subjects (28 MS patients, and 2 controls) were examined separately by the two neuroradiologists, each blinded to the results obtained by the other one. The operators were not blinded for clinical status as healthy subjects or MS patient, but were blinded for MS subgroups. The results were then compared to evaluate the inter-observer concordance.

IJVs and VVs were studied in B-mode. To define the stenosis the vessel calibre was reduced more than 50%. The cross-sectional area (CSA) of IJVs and VVs were measured in horizontal plane, avoiding any vessel compression. The CSA of right and left IJVs were measured at their middle tract, and under valve plane in both clinostatism and seated position. The CSA of right and left VVs were measured under the valve plane in both clinostatism and seated position. Exact angle correction of Doppler frequencies was achieved by adjusting the angle between the Doppler bean and the longitudinal axes of the vessel.

CVF of IJVs and VVs was calculated from the time average velocity (TAV) and the CSA of the vessel (CVF = CSAxTAV). TAV was measured over a minimum of the three cardiac cycles at the end of the expiratory phase. [3], [13], [16] CVF of each vein was calculated in both clinostatism and seated position. The sum of all the venous flows was then calculated in clinostatism (CVFC) and in seated position (CVFS). The difference between the CVFC and CVFS (Δ value = CVFC-CVFS) has been named ΔCVF, and included in the analysis. CBF was calculated in the same way, based on the TAV and CSA of internal carotid and vertebral arteries, only in clinostatism. [1]–[3]

A statistical analysis was performed between the groups (healthy subjects and MS patients) evaluating the Δ value (positive or negative) and characteristics of CVF at 0° and 90°, Δ CSA of IJVs at 0°and 90°, CBF, and clinical conditions including EDSS, disease duration, age, gender, NHPT, and T8.

Statistical analysis was performed by Fisher's exact test, non parametric Mann-Whitney U test, ANOVA Kruskal-Wallis test, and non linear correlation (correntropy coefficient).[31]–[35] The data showed a non Gaussian and a non symmetric behaviour, when collected by groups they even resulted in small sets of observations. The statistical analysis was performed by means of non parametric tests, namely Fisher's exact test for assessing the significance of differences in contingency tables, Mann-Whitney test and ANOVA Kruskal-Wallis test for comparing the medians of two or more groups, respectively. The level of interaction between the ΔCVF and the other variables was determined by the correntropy coefficient, which is a measure of correlation suitable for nonlinear, non Gaussian data, ranging in the interval [−1, +1], sensitive to higher order moments of the signals. More specifically, the correntropy function can be seen as a generalized correlation function in the space (feature space) spanned by the nonlinear mapping (kernel mapping) of the original data.[32]–[35] Unlike the Pearson's coefficient, the correntropy coefficient will produce a non zero value for two uncorrelated but not independent variables and in the context of generalized synchronization, the correntropy coefficient is able to characterize both higher order relationship and nonlinearity between interacting systems. The geometrical elucidation of the correntropy coefficient essentially is that it computes the cosine of the angle between two nonlinear transformed vectors in the higher dimensional feature space. Thus, only if the two compared series are independent, their vectors in the higher dimensional feature space will be orthogonal, and so the cosine of their angle is equal to zero. For this reason, we can recognize the correntropy coefficient as a measure of dependence (conveyed either by a linear relationship or by a nonlinear one).

## Results

29 MS patients were undergoing a Disease Modifying Therapy at the time of CDS evaluation. EDSS, NHPT, and T8 results of MS patients are shown in Table 1.

**Table 1:** MS clinical summary data.

	EDSS	Disease Duration (months)	NHPT Right (sec.)	NHPT Left (sec.)	T8 (sec.)
Average	2.962	127.8	28.57	29.27	9.49
Std	1.93	96.42	34.52	27.71	13.81

In the first 30 examined subjects, the concordance between the two different blinded observers was 86,6%.

15 IJVs stenosis were demonstrated in the MS patients group.

**Figure 1:**
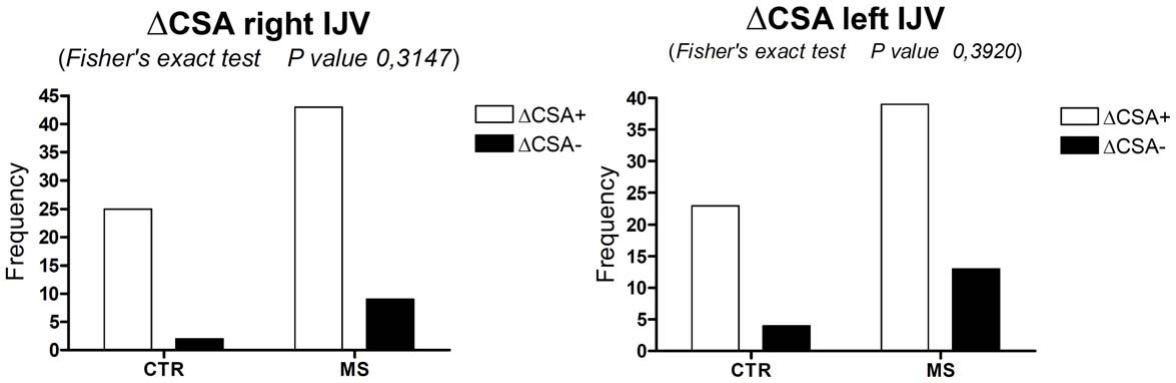
Fisher's exact test applied to ΔCSA of right and left IJV within the two study groups: patients with MS (MS) and healthy subjects (CTR). The differences were not significant.

The ΔCSA of the right IJV at 0° and 90° position was positive in 43 out of 52 (82.69%) patients, and negative in 9 (17.3%). In the control group, ΔCSA was positive in 25 out of 27 (92.59%) subjects. These differences were not significant (p = 0.3147, Fisher's exact test). The ΔCSA of the left IJV at 0° and 90° position was positive in 39 out of 52 (75%) patients and negative in 13 (25%). In the control group, ΔCSA was positive in 23 out of 27 (85.2%) subjects. These differences were not significant (p = 0.3920, Fisher's exact test) (Figure 1).

**Figure 2:**
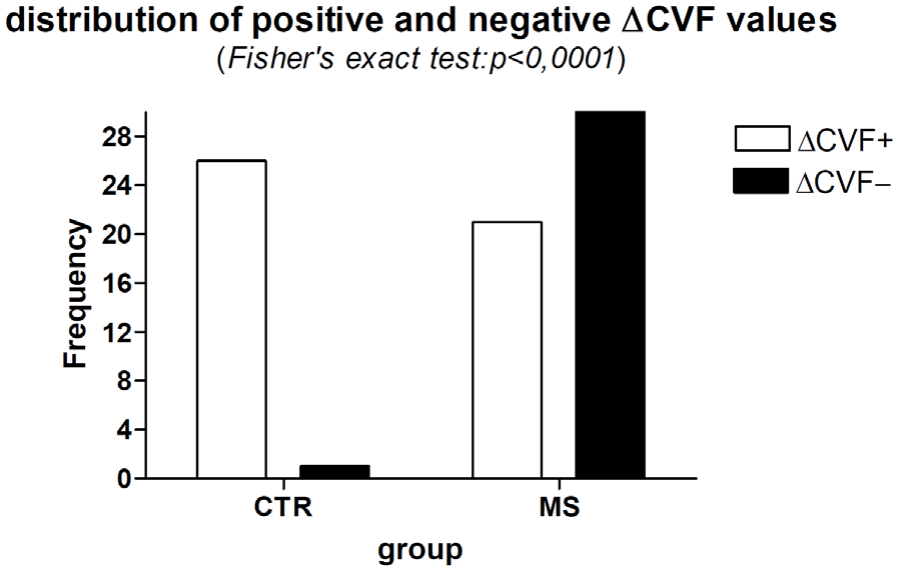
Graphic corresponding to the contingency table (Tab.II). Fischer exact test resulted in highly significance (p<0.0001). The sign of the ΔCVF is associated to specific Group: negative value is related to pathologic condition.

**Figure 3:**
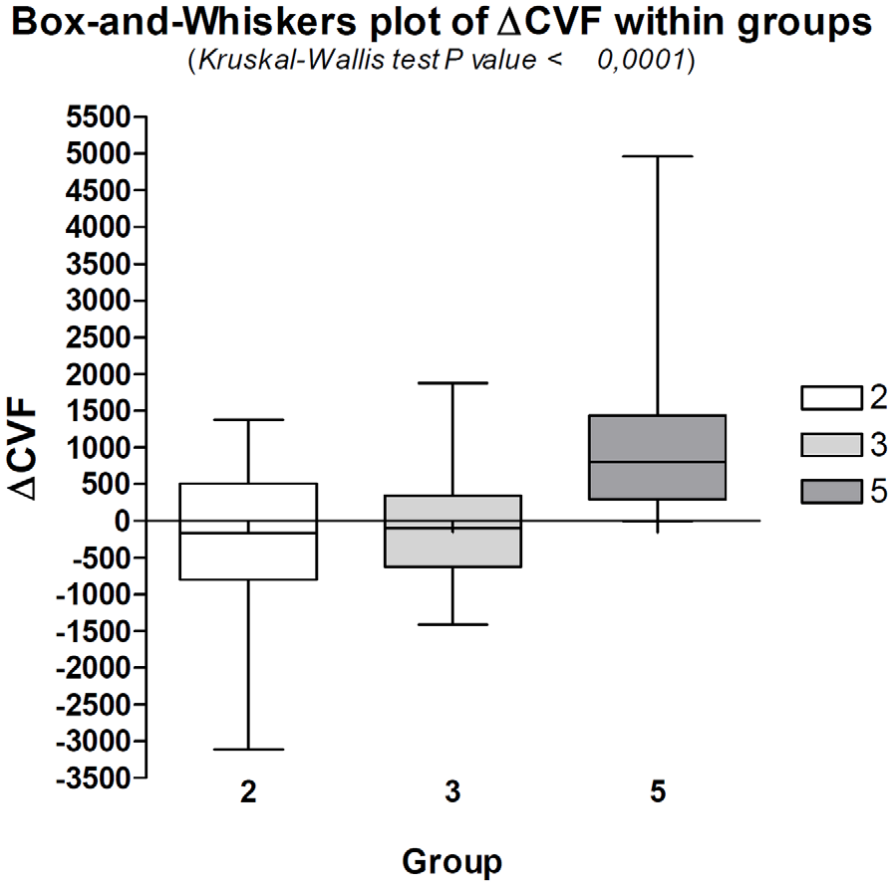
Box-and-Whiskers plot of ΔCVF within prevalent subgroups of MS and controls. Group 2: RRMS; Group 3: SPMS; Group 5: controls. There is a significant difference between Group 5 and the Groups 2 and 3.

Fisher's exact test demonstrated that a negative ΔCVF is significantly associated with MS (p<0.0001), while a positive ΔCVF is correlated to normal physiological condition (Figure 2, Table 2). Non parametric ANOVA Kruskal-Wallis test resulted significant (p<0.0001) in the analysis of ΔCVF among Groups 2, 3, and 5 (Figure 3). Post hoc evaluation carried out by means of the Dunn's multiple comparison test which showed a significant difference between Groups 2 vs Group 5 (p<0.001), and Group 3 vs Group 5 (p<0.01), while there was no difference between Group 2 and Group 3. The negative ΔCVF was 61.3% in Groups 2 and 52.9% in Group 3: the Fisher's exact test showed that this difference was not statistically significant (p = 0.76). Groups 1 and 4 were not considered in the ANOVA test for their limited numbers. The ΔCVF negative value was absent in 96.3% of controls, while was present in 59.6% of MS patients.

**Table 2:** Contingency table.

Groups	ΔCVF positive value	ΔCVF negative value	Total
CTR n°	26	1	27
MS n°	21	31	52
Total n°	47	32	79

CTR = healthy subjects; MS = patients with MS; ΔCVF = CVF in clinostatism - CVF in the seated position.

**Figure 4:**
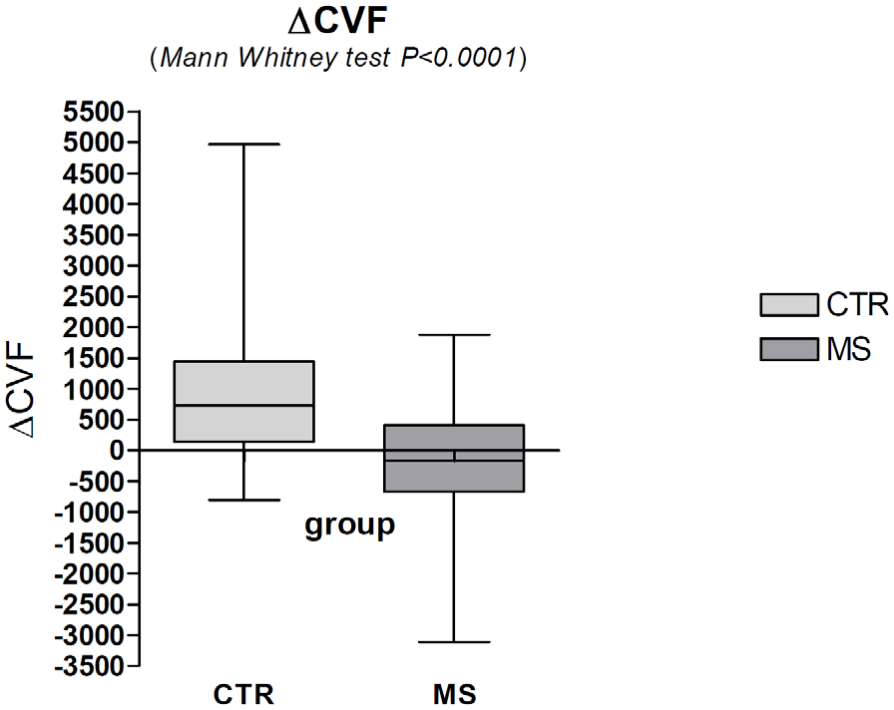
Mann-Whitney test demonstrates a significant difference (p<0.0001) in the comparison of the ΔCVF values between the group of healthy subjects (CTR) and the aggregated groups (1,2,3,4) of patients (MS).

Mann-Whitney test comparison of the median of MS patients and controls groups resulted statistically significant (p<0.0001) (Figure 4).

**Figure 5:**
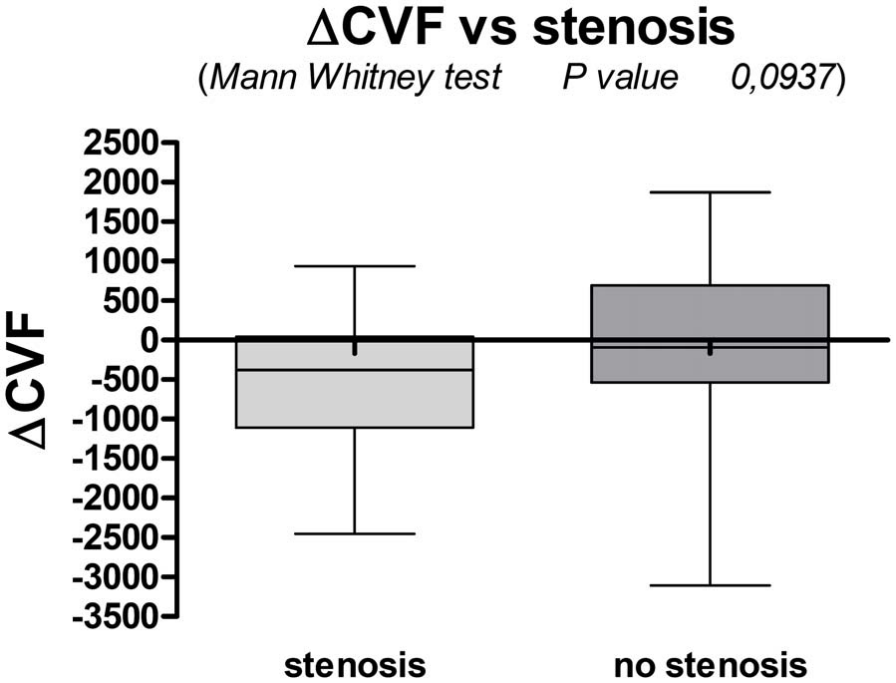
The ΔCVF was not statistically correlated with the group of patients with stenosis (p = 0.0937, Mann-Whitney test).

Negative values of the ΔCVF was not statistically correlated with the group of patients with stenosis (p = 0.0937, Mann-Whitney test) (Figure 5).

No CBF difference was found between controls and MS patients, as reported in the Mann-Whitney test results (p = 0.3684).

Negative or positive values of the ΔCVF were not correlated to any other clinical parameter as age, gender, disease duration, EDSS, NPTH, and T8.

Group 5 showed a positive correntropy coefficient in the clinostatism position, while MS patients showed an anticorrelation between ΔCVF and the flow of the IJVs in measurements obtained in the seated position (Table 3).

**Table 3:** Correntropy coefficients between ΔCVF and BVF (IJVs and VVs) data.

	MS	CTR
η(ΔCVF,BVF VV C)	0.1523	0.1587
η(ΔCVF,BVF VV O)	0.0823	−0.0958
η(ΔCVF,BVF IJV C)	0.1307	0.5374
η(ΔCVF,BVF IJV O)	−0.2953	−0.1264

The interaction between ΔCVF and BVF (IJVs and VVs) for MS group and CTR group behaved almost in the same way at the VVs flow recording, as revealed by the magnitudes of the correntropy coefficients (first and second rows of the table).The variable ΔCVF seemed to be mainly associated to the BFV variable measured at the IJVs. The healthy subjects showed a positive correntropy coefficient in the clinostatism position (clinostatism), while for the patients there was an anticorrelation between ΔCVF and BVF of the IJVs in the seated position measurement.

## Discussion

Anatomical findings support that IJVs are the principle outflow pathway for intracranial blood in clinostatism, while in an erect position the venous drainage is partially compensated by VVs. The IJVs venous drainage is physiologically reduced in a seated position. Thus, the CVF in clinostatism should be higher than the CVF in seated position. CVF has usually been calculated by CDS such as for the cerebral arterial inflow. It is common knowledge that sonographic CVF measurement presents technical limitations in the exact CSA when the IJV distal tract has a deep intrathoracic position, as well as during different respiratory and cardiac phases. Moreover, the reliability of sonographic examination highly depends on the skill and experience of the examiner. In this study, the evaluation of the blinded interobservers on a cohort of patients and controls resulted in a highly acceptable concordance.

Angiography and venography are considered the gold-standard techniques for the evaluation of vascular pathology, and most notably for the venous system, since they can demonstrate vessels morphology, turbulent flow, stenosis, and valves. However, both angiography and venography do not allow to obtain a correct dynamic study during postural changes neither to evaluate quantitative data of CVF.

Our study considered the distal IJVs and VVs in order to obtain the effective venous outflow, in presence or not of stenosis further up the distal tract. We observed that the negative value of the ΔCVF is an important finding which is able to detect a statistically significant correlation with a pathologic condition. The negative value of the ΔCVF has an incidence of 59.6% in patients with MS, while in healthy controls the ΔCVF is nearly always positive (96.3%).

This CDS parameter might be correlated to an abnormal venous return. The negative value of ΔCVF suggests a higher venous outflow in patients with MS in a seated position, instead of a lying position. The positive value of ΔCVF is in accordance with a normal cerebral venous return. In fact, IJVs are usually the major cerebral venous drain in clinostatism, while in the seated position they are liable to collapse. This seems to suggest an abnormal cerebral venous drainage in the majority of MS patients, especially in seated position. We can rule out a technical artifact, since the frequency is not the same in the control group. Nowadays, in the literature there is no agreement about the value of the ΔCSA for MS.[13], [19]–[20] Concurring with Doepp's study, we demonstrated no significant correlation between ΔCSA and patients with MS, or the presence of stenosis. While, the ΔCVF negative value seems to correlate to a pathologic condition. The sign of the ΔCVF value certainly has an hemodynamic significance, however there was no statistically correlation between the ΔCVF value and age, duration of the disease, EDSS, NHPT, and T8. Prevalence of negative ΔCVF in patients with relapsing remitting (Group 2) and secondary progressive (Group 3) MS didn't differ significantly from each other. This does not help to explain a specific clinical value of this parameter. Only IJV outflow in the seated position was responsible for the negative sign of ΔCVF value. We may hypothesize a different hemodynamic condition in MS patients, despite the absence of relationship with IJV stenosis or an ongoing venous congestion. The patients with MS might have a venous congestion evident only in clinostatism and an anomalous venous outflow only in a seated position. Intraluminal abnormalities of IJV might be hypothesized, since valve abnormalities or thin intraluminal septum could explain the reduced IJV outflow in supine position. However, an intraluminal abnormality should reduce the outflow in both supine and seated position, and doesn't explain why the IJV outflow in a seated position is higher than in a supine position. Other possible hemodynamic explanations include: i) a valve incompetence of IJVs, ii) no efficient SEVs outflow as an additional drainage pathway in the seated position, possibly caused by stenosis of the vertebral veins or incompetence of vertebral plexus when seated, iii) any pathology affecting azygous, hemyazygous, or venous system, and iiii) a vascular dysregulation from the release of vasoactive substances. [36]–[37] Finally, an abnormal compliance of venous vessel wall might be due to MS affecting the autonomous nervous system. [38]

The limitations of our study are the partial limited number of MS patients and MS subgroups, and that the operators were not blinded to the clinical status as healthy or MS patient. However, the operators were blinded to the MS subgroups.

Recently, numerous authors have demonstrated different results about CCSVI. The five parameters of Zamboni identify CCSVI and classify two different groups of patients: those eligible for venography and those who are not. [18] Zivadinov demonstrated the prevalence, sensitivity and specificity of sonographic parameters in CCSVI. [39] Baracchini et al. demonstrated that even if sonographic parameters of CCSVI are present in 16% of MS patients and one or more abnormal extracranial ultrasound findings were observed in 52% of MS patients, venography doesn't reveal venous system pathology or vessel anomalies. [40]–[41] These studies and others suggest the need for a multimodal approach for this topic. [42] The purpose of our study was not to verify the presence of CCSVI and its sonographic parameters, but to demonstrate if there is a different quantitative value of haemodynamic alterations between MS patients and healthy controls. Ongoing studies will add whether or not negative ΔCVF has a relationship to brain damage at MR imaging, cerebral hypoperfusion,[42] and CCSVI.[14], [18]–[19].

In conclusion, the quantitative evaluation of the majority of CVF may be reproducible like the arterial blood flow. Our study has shown that the quantitative evaluation of CVF and its postural dependency can have a relevant hemodynamic significance. We propose ΔCVF as a new sonographic parameter to demonstrate an anomalous cerebral venous drainage, although there is not enough data to understand its clinical relevance. In the future, patients with other neurological pathologies and a greater number of MS patients and MS subgroups will be enrolled.
